# A novel heterozygous *HTRA1* mutation in an Asian family with CADASIL‐like disease

**DOI:** 10.1002/jcla.24174

**Published:** 2021-12-23

**Authors:** Hua Cao, Jiahui Liu, Wen Tian, Xiaofei Ji, Qi Wang, Siyu Luan, Xiang Dong, Huijie Dong

**Affiliations:** ^1^ Department of Neurology First Affiliated Hospital of Dalian Medical University Dalian China; ^2^ Department of Cardiology Second Affiliated Hospital of Dalian Medical University Dalian China

**Keywords:** CADASIL‐like disease, heterozygous mutation, *HTRA1*

## Abstract

**Background:**

*HTRA1* gene mutations are related to the pathogenesis of cerebral autosomal recessive arteriopathy with subcortical infarcts and leukoencephalopathy (CARASIL). However, heterozygous *HTRA1* mutations at specific sites can also lead to rare autosomal dominant cerebral artery disease (CADASIL‐like disease). To date, 28 heterozygous mutations in the *HTRA1* gene have been reported to be related to CADASIL‐like diseases. Only one case of this disease was caused by a heterozygous mutation of c.497G>T in exon 2 of the *HTRA1* gene.

**Methods:**

In this case, we report on an Asian family with CADASIL‐like disease caused by a heterozygous mutation of c.497G>T in exon 2 of the *HTRA1* gene. The clinical and imaging characteristics of the proband were summarized, and gene mutations were verified by whole‐exome sequencing (WES) and direct Sanger sequencing.

**Results:**

The result of the gene sequencing showed a heterozygous missense mutation at the c.497G>T locus of the *HTRA1* gene in the proband of one sick family member, resulting in a change in amino acid (p.arg166leu).

**Conclusion:**

This is the first reported pathogenic mutation at the c.497G>T locus of the *HTRA1* gene in an Asian population. It provides an important theoretical basis for the specific gene‐based diagnosis and treatment of CADASIL‐like diseases.

## Graphical abstract

CADASIL‐like disease is a rare hereditary cerebrovascular disease, which is related to the heterozygous mutation of HTRA1 gene. The heterozygous mutation in this gene is just like the damaged track shown in the figure, inducing the train to tilt to the disease area. In this paper, we report for the first time an Asian pedigree of CADASIL‐like disease caused by the mutation of c.497G>T locus in exon2 of HTRA1 gene. It provides an important theoretical basis for the specific gene‐based diagnosis and treatment of CADASIL‐like diseases.

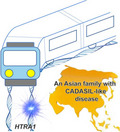

## 1 INTRODUCTION

Cerebral small vessel disease (CSVD) refers to lesions of the cerebral arterioles, venules, and capillaries caused by various causes.^1^ Clinical manifestations of this type of disease are cognitive decline, abnormal gait, decreased executive function, mental symptoms, etc.^2^ CSVD can be divided into sporadic and hereditary. Sporadic CSVD is mainly related to age, hypertension, diabetes, smoking, and other risk factors.^3^ Hereditary CSVD accounts for approximately 5% of all cerebrovascular diseases caused by gene mutations, including cerebral autosomal dominant arteriopathy with subcortical infarcts and leukoencephalopathy (CADASIL), cerebral autosomal recessive arteriopathy with subcortical infarcts and leukoencephalopathy (CARASIL), Fabry disease, retinal vasculopathy with cerebral leukoencephalopathy (RVCL), COL4A1‐related diseases, etc.^4^ Among the known pathogenic genes of CSVD, the *HTRA1* gene is related to the pathogenesis of CARASIL. However, heterozygous *HTRA1* mutations at specific sites can also lead to rare autosomal dominant cerebral artery disease (CADASIL‐like disease).^5^

The *HTRA1* gene is located on the long arm of chromosome 10 and consists of nine exons. This gene encodes the synthesis of HTRA serine protease. The encoded protein contains four functional domains and participates in various pathological processes, including arthritis, cancer, cerebrovascular disease, and neurodegenerative diseases.^6^ To date, 45 *HTRA1* gene mutations related to CSVD have been reported, including 23 types of CADASIL‐like diseases caused by heterozygous *HTRA1* mutations. Five other types of heterozygous *HTRA1* mutations were found to be associated with CARASIL and CADASIL‐like disease.^7^ There was only one case of CADASIL‐like disease caused by c.497G>T heterozygous mutation in exon 2 of the *HTRA1* gene which has been reported to date.^5^

Herein, we report a case of an Asian family with CADASIL‐like disease caused by a c.497G>T heterozygous mutation in exon 2 of the *HTRA1* gene. We analyzed the clinical features, imaging features, and gene sequencing results of the proband and drew the disease pedigree map to provide a clinical basis for the diagnosis of this disease.

## 2 MATERIALS AND METHODS

### 2.1 Patient and families

**FIGURE 1:**
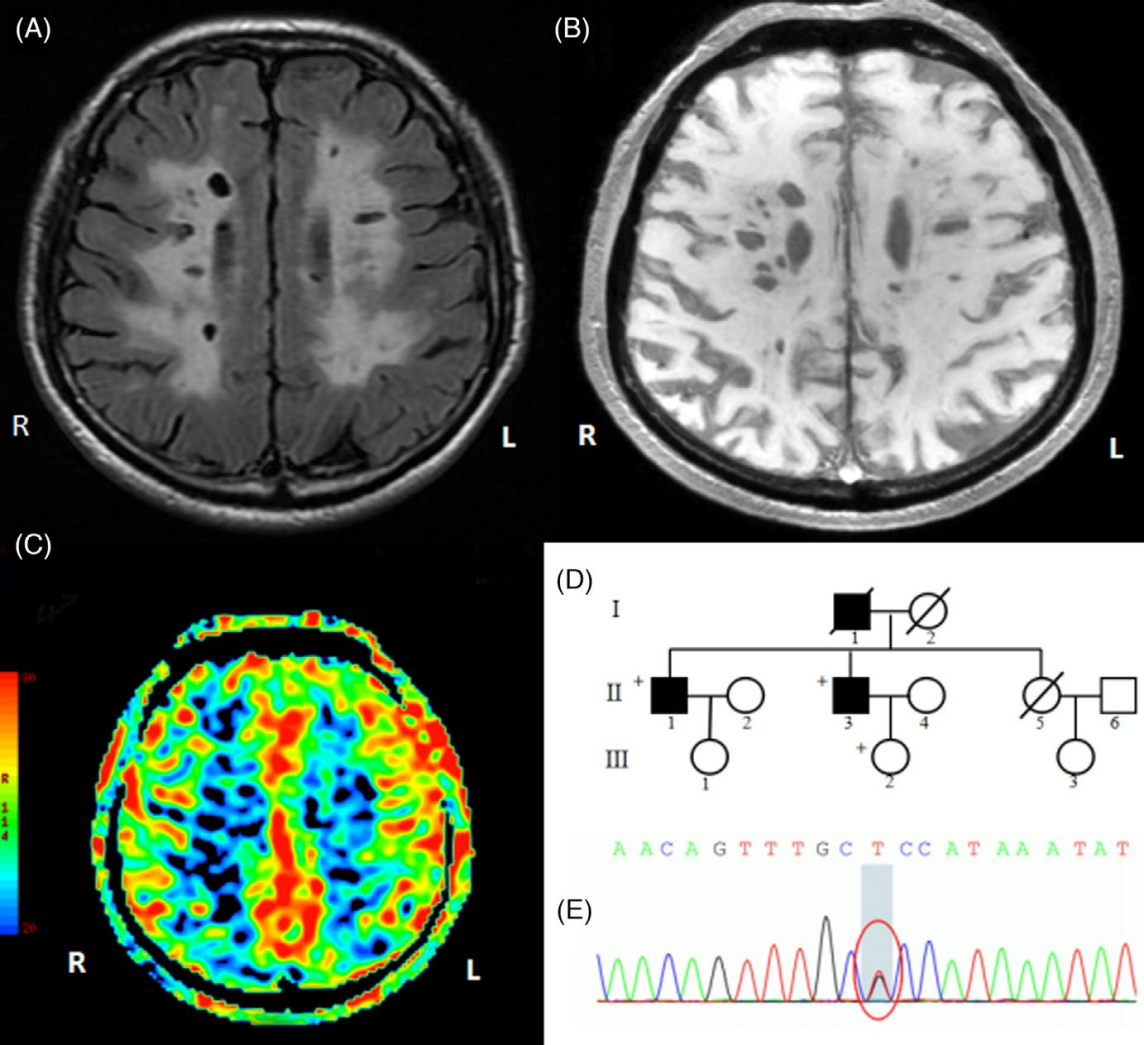
Brain magnetic resonance examination and genotype results of the proband (II‐1). (A) and (B): Severe diffuse leukoencephalopathy in deep white matter; (C): Cerebral blood flow decreased in deep white matter; (D) Disease pedigree map; (E): Sequence chromatograms of the heterozygous mutation *HTRA1* c.497G>T. Square, male; circle, female; full black filled symbol, clinically and magnetic resonance imaging (MRI)‐proven affected individuals; empty symbol, clinically healthy relatives; plus sign, mutation carriers

We present a patient with CADASIL‐like disease, who was admitted to the Department of Neurology, First Affiliated Hospital of Dalian Medical University, China. Peripheral blood samples from the proband (II‐1 in Figure 1d) and one sick family member (II‐3 in Figure 1d) were collected for investigation. Other immediate relatives of the proband refused the gene detection. The study was approved by the Ethics Committee of the First Affiliated Hospital of Dalian Medical University and was conducted following the recommendations of the Declaration of Helsinki. Written informed consent was obtained from all patients. The disease pedigree map is shown in Figure 1d.

### 2.2 Mutation analysis

Whole‐exome sequencing (WES) was performed on DNA extracted from peripheral blood samples. After fragmenting the genomic DNA, ligating the paired‐end adaptors, amplifying the DNA, and purifying the amplified products, all human exons and the 50 bp regions of their adjacent introns were captured using the xGen Exome Research Panel (Integrated DNA Technologies). The DNA library was constructed following amplification, purification, and capture and then sequenced on a HiSeq sequencing platform (Illumina). We used NextGENe v2.3.4 and the laboratory's own scripts to obtain the annotation information, including the conserved nucleotide bases and amino acids, predictions of the biological functions, and frequency of the normal populations (1000 Genomes Project, Exome Aggregation Consortium (ExAC), database of single nucleotide polymorphisms (dbSNP), and locus‐specific databases), as well as data from the Human Genome Mutation Database, ClinVar, and Online Mendelian Inheritance in Man. Polymerase chain reaction (PCR) amplification and direct Sanger sequencing were used to verify the suspicious gene mutation sites detected by WES. The amplified PCR products of the *HTRA1* gene were visualized on a 2% agarose gel. To discover harmful mutations, BLAST (https://blast.ncbi.nlm.nih.gov/) was used to align the sequence data with the *HTRA1* reference DNA sequence.^8^, ^9^

## 3 CASE DESCRIPTION

### 3.1 Disease history

The proband was a 59‐year‐old Chinese Han male with intellectual decline, unstable walking, poor urinary control for 6 months, and acute onset weakness of the right lower limb for 1 month. During the course of the disease, symptoms, such as headache, trichomadesis, nausea, vomiting, unclear speech, blurred vision, dysdipsia and dysphagia, limb twitching, dysuria, or unconsciousness, were not present.

### 3.2 Past history and family history

The proband had a history of cerebral infarction for 2 years. There was no history of hypertension, diabetes, coronary heart disease, or smoking. The father had a history of multiple cerebral infarction and dementia and died in stroke 15 years ago. The 55‐year‐old brother of the proband also had cerebral infarction for 1 year. Head magnetic resonance (MR) at onset showed multiple demyelinating changes in cerebral white matter.

### 3.3 Physical examination of the nervous system

The proband was conscious, fluent, and hypomnetic. The cranial nerves were normal. The muscle strength of the left upper limb and both lower limbs and the muscle strength of the right upper limb were scored 4 and 5, respectively. However, the bilateral point‐to‐point test was not accurate. The upper and lower limb tendons showed overactivity. The Babinski and Chaddock signs were bilateral positive.

### 3.4 Additional examinations

Head magnetic resonance (MR) showed the following results: (1) multiple lacunar cerebral infarction and demyelination of white matter in the bilateral semioval center, bilateral paraventricular and bilateral basal ganglia, left thalamus, and right cerebellar hemisphere (Figure 1a); (2) multiple microbleeds in the bilateral cerebral hemispheres, basal ganglia, thalamus, brainstem, and cerebellum (Figure 1b); and (3) decreased perfusion in the right hemisphere (Figure 1c).

### 3.5 Genetic analysis

Genomic DNA was extracted from peripheral blood samples of the proband. Spectrophotometric analysis was used to measure the concentration and purity of DNA samples.^10^, ^11^ One heterozygous missense variation, c.497G>T, was identified in exon 2 of the *HTRA1* gene by WES. The WES of his sick brother with symptoms revealed the same heterozygous variant c.497G>T (p. Arg166Leu) in the *HTRA1* gene. The quality control data for the WES are listed in Table 1. To further verify the results of the gene test, PCR amplification and direct Sanger sequencing were used to detect the variant in exon 2 of the *HTRA1* gene. The sequences of primers used were as follows: F‐5′‐CTTACCTGGGTGGGCACTC‐3′ and R‐5′‐TGTTCTAAGGGAGACACACTTATC‐3′. We verified a definite gene mutation of c.497G>T in exon 2 of *HTRA1* (Figure 1e).

**TABLE 1:** Quality control data of whole‐exome sequencing

Total	
Raw_data (Mb)	2407.42
Clean_data (Mb)	2232.11
Aligned (%)	99.88
Initial bases on target	1929086
Base covered on target	1925806
Coverage of target region	99.80%
Total effective yield (Mb)	1736.66
Effective sequence on target (Mb)	850.98
Fraction of effective bases on target	49.00%
Average sequencing depth on target	441.13
Fraction of target covered with at least 4×	99.40%
Fraction of target covered with at least 10×	98.70%
Fraction of target covered with at least 20×	97.60%
Duplication rate (%)	21.25

## 4 DISCUSSION

The *HTRA1* gene mutations often lead to the pathogenesis of CARASIL. Most parents of patients have a history of close relative marriage. It is a non‐hypertensive cerebrovascular disease that occurs in youth. Clinically, it is characterized by progressive nervous system damage, including early‐onset recurrent stroke, subcortical dementia, affective disorders, gait disorder, and extraneural characteristics, including hair loss and spondylosis.^12^ However, heterozygous mutations in the *HTRA1* gene also can lead to rare CADASIL‐like disease, which is an autosomal dominant disease. Studies found that compared with typical CARASIL, patients with CADASIL‐like disease had a later onset age, a higher proportion of vascular risk factors, a lighter and relatively slow clinical progress, and a lower incidence of extraneurological symptoms, such as early‐onset spondylosis and hair loss.^7^, ^13^ Due to the late‐onset and the high proportion of vascular risk factors, the family history of this disease is often ignored. Therefore, for patients with CSVD with unknown etiology, the *HTRA1* gene screening and head magnetic resonance imaging (MRI) imaging should be considered to determine whether it has a clear family history. The head MRI of the patient with CADASIL‐like disease has typical characteristics of CSVD, such as white matter lesions, subcortical multiple lacunar infarcts, and intracranial microbleeds.^3^ CADASIL‐like disease is mainly characterized by intimal thickening, adventitia fibrosis, degeneration and loss of smooth muscle cells, and stratification and division of the inner elastic layer of small vessels in the central nervous system. However, there are no osmiophilic particles on the surface of the vascular smooth muscle under an electron microscope. Therefore, a skin biopsy can provide a basis for differentiation from typical CADASIL.^14^ With the development of WES technology, gene detection has played an important role in clinical diagnosis, treatment, and scientific research.

In this study, a heterozygous mutation caused by c.497G>T in an Asian family was reported. The clinical features of the proband were similar to those of the same mutation reported in a previous study, such as history of stroke and cognitive impairment.^5^ Similar severe diffuse leukoencephalopathy in the deep white matter can be seen on MRI examination. We also observed a significant decrease in cerebral blood flow in white matter lesions.

To date, the pathogenesis of this disease is not fully understood, and there are no special treatment methods. This study focuses mainly on symptomatic support treatment. At the same time, it provides genetic counseling for patients to guide them in avoiding relevant risk factors. It is also recommended to further carry out relevant evaluation and gene screening for the families of patients with a confirmed diagnosis of CSVD caused by genetic variation.^6^ With the increasing basic and clinical research on the pathogenesis of this disease and an in‐depth understanding of the pathophysiological mechanism, it is necessary to find potential therapeutic targets and provide more theoretical support for its clinical diagnosis and treatment.

## 5 CONCLUSION

Our findings of a heterozygous c.497G>T mutation in this Asian family provide novel evidence for the *HTRA1* mutation in CADASIL‐like disease. This finding will improve genetic counseling for both relatives of CARASIL patients and carriers of *HTRA1* variants with sporadic CSVD.

## CONFLICT OF INTEREST

The present study does not have any conflict of interest.

## Supporting information

Supplementary Material: Partial mutation data of HTRA1 gene detected by WES

## Data Availability

Data supporting the findings of this study are available from the corresponding author upon reasonable request.

## References

[1] Pantoni L . Cerebral small vessel disease: From pathogenesis and clinical characteristics to therapeutic challenges. Lancet Neurol. 2010;9(7):689‐701. 20610345 10.1016/S1474-4422(10)70104-6

[2] Smith EE , O'Donnell M , Dagenais G , et al. Early cerebral small vessel disease and brain volume, cognition, and gait. Ann Neurol. 2015;77(2):251‐261. 25428654 10.1002/ana.24320 PMC4338762

[3] Li Q , Yang Y , Reis C , et al. Cerebral small vessel disease. Cell Transplant. 2018;27(12):1711‐1722. 30251566 10.1177/0963689718795148 PMC6300773

[4] Søndergaard CB , Nielsen JE , Hansen CK , Christensen H . Hereditary cerebral small vessel disease and stroke. Clin Neurol Neurosurg. 2017;155:45‐57. 28254515 10.1016/j.clineuro.2017.02.015

[5] Verdura E , Hervé D , Scharrer E , et al. Heterozygous *HTRA1* mutations are associated with autosomal dominant cerebral small vessel disease. Brain. 2015;138(Pt8):2347‐2358. 26063658 10.1093/brain/awv155

[6] Tikka S , Baumann M , Siitonen M , et al. CADASIL and CARASIL. Brain Pathol. 2014;24(5):525‐544. 25323668 10.1111/bpa.12181 PMC8029192

[7] Uemura M , Nozaki H , Kato T , et al. HTRA1‐related cerebral small vessel disease: a review of the literature. Front Neurol. 2020;11:545. 32719647 10.3389/fneur.2020.00545 PMC7351529

[8] Dai Y , Liang S , Dong X , et al. Whole exome sequencing identified a novel DAG1 mutation in a patient with rare, mild and late age of onset muscular dystrophy‐dystroglycanopathy. J Cell Mol Med. 2019;23(2):811‐818. 30450679 10.1111/jcmm.13979 PMC6349151

[9] Zheng Y , Xu J , Liang S , et al. Whole exome sequencing identified a novel heterozygous mutation in HMBS gene in a Chinese patient with acute intermittent porphyria with rare type of mild anemia. Front Genet. 2018;9:129. 29731767 10.3389/fgene.2018.00129 PMC5920022

[10] Han P , Wei G , Cai K , et al. Identification and functional characterization of mutations in LPL gene causing severe hypertriglyceridaemia and acute pancreatitis. J Cell Mol Med. 2020;24(2):1286‐1299. 31901151 10.1111/jcmm.14768 PMC6991700

[11] Zhang R , Chen S , Han P , et al. Whole exome sequencing identified a homozygous novel variant in CEP290 gene causes Meckel syndrome. J Cell Mol Med. 2020;24(2):1906‐1916. 31840411 10.1111/jcmm.14887 PMC6991682

[12] Di Donato I , Bianchi S , Gallus GN , et al. Heterozygous mutations of *HTRA1* gene in patients with familial cerebral small vessel disease. CNS Neurosci Ther. 2017;23(9):759‐765. 28782182 10.1111/cns.12722 PMC6492684

[13] Liu JY , Zhu YC , Zhou LX , et al. HTRA1‐related autosomal dominant cerebral small vessel disease. Chin Med J (Engl). 2020;134(2):178‐184. 33109952 10.1097/CM9.0000000000001176 PMC7817319

[14] Ito J , Nozaki H , Toyoshima Y , et al. Histopathologic features of an autopsied patient with cerebral small vessel disease and a heterozygous *HTRA1* mutation. Neuropathology. 2018;38(4):428‐432 10.1111/neup.12473 29797751

