# Supplementary material for: A novel heterozygous HTRA1 mutation in an Asian family with CADASIL‐like disease
Source: J Clin Lab Anal. 2021 Dec 23;36(2):e24174. doi: 10.1002/jcla.24174 (PMC8841136; doi:10.1002/jcla.24174)
Supplement: Supplementary file 1 — Supplementary Material: Partial mutation data of HTRA1 gene detected by WES [file JCLA-36-e24174-s001.docx]

#Chrom Start Stop Refer Call Zygosity VarType Filter ADepth ARatio DP PL NeighborGID PhasedGID DeNovo MutationName GeneSym EntrezGeneID Transcript cHGVS Protein pHGVS Strand PrimaryTag FunctionName gHGVS StandardMutation ExInID cDNAPos AAPos CDSPos AnnoTag LocalPanelAC RepeatTag MapLoc OMIMDisease OMIMGeneID OMIMInheritance OMIMPhenoIDs OMIMStat ClinVarAF ClinVarAlleleOrigin ClinVarDisease ClinVarID ClinVarStatus ClinicalSignificance HGMDComments HGMDDisease HGMDID HGMDPmid HGMDPred HGMDSupport CGDAllelicCondition CGDCondition CGDInheritance CGDInterventionCategories CGDManifestationCategories CGDReferences CosmicHistology CosmicID CosmicMutName CosmicMutStat CosmicSite GWASTrait dbSNPAF rsID ExACAC ExACAF ExACEASHetAC ExACEASHomAC ExACFilter gnomAD_exome_AFR_AC gnomAD_exome_AFR_AF gnomAD_exome_ALL_AC gnomAD_exome_ALL_AF gnomAD_exome_AMR_AC gnomAD_exome_AMR_AF gnomAD_exome_ASJ_AC gnomAD_exome_ASJ_AF gnomAD_exome_EAS_AC gnomAD_exome_EAS_AF gnomAD_exome_FIN_AC gnomAD_exome_FIN_AF gnomAD_exome_NFE_AC gnomAD_exome_NFE_AF gnomAD_exome_OTH_AC gnomAD_exome_OTH_AF gnomAD_exome_SAS_AC gnomAD_exome_SAS_AF ESP6500_AA_AC ESP6500_AA_AF ESP6500_ALL_AC ESP6500_ALL_AF ESP6500_EA_AC ESP6500_EA_AF TGPAC TGPAF TGP_CHB_AF TGP_CHS_AF TGP_EAS_AF PVFDAF CGFreDBAF CGFreDBTag inhouse_AF inhouse_AN inhouse_Status CADD CADD_phred DANN_score FATHMMPred FATHMMScore GERP_RS LRTPred LRTScore MCAP_Score MetaLRPred MetaLRScore MetaSVMPred MetaSVMScore MutationAssessorPred MutationAssessorScore MutationTasterPred MutationTasterScore PROVEANPred PROVEANScore Polyphen2HumDivPred Polyphen2HumDivScore Polyphen2HumVarPred Polyphen2HumVarScore SIFTPred SIFTScore VEST3_score fathmmPred fathmmScore fitConsScore PfamID PfamName PhylopPlacentalmammals PhylopPrimates PhylopVertebrate dpsi_max_tissue dpsi_zscore InterVar hgmdinfo

chr10 124248442 124248442 G T Het snv PASS 206 0.43 476 "4959,0,7155" . . . NM_002775.4(HTRA1): c.497G>T (p.R166L) HTRA1 5654 NM_002775.4 c.497G>T NP_002766.1 p.R166L + . missense g.124248441G>T . exon2 625 166 497 CDS . . 10q26.13 "Macular degeneration, age-related, neovascular type" 602194 AR 600142(MK)|610149(SK) P . germline "Cerebral_arteriopathy,_autosomal_dominant,_with_subcortical_infarcts_and_leukoencephalopathy,_type_2" 221228 no_assertion_criteria_provided Pathogenic R166L "Cerebral small vessel disease, autosomal dominant" CM157924 26063658 DM NULL . Cerebral autosomal recessive arteriopathy with subcortical infarcts and leukoencephalopathy (CARASIL) AR General Cardiovascular; Dermatologic; Neurologic 17053108; 17053109; 17884985; 17568988; 18511946; 19387015; 20437615; 21115960; 21482952; 22900900 . . . . . . . . . . . . . . . . . . . . . . . . . . . . . . . . . . . . . . . . . . . . . . . . 7.674145 35 0.998702835 D -1.56 5.42 D 0 0.164403513 D 0.7599 D 0.6791 M 2.845 D 1 D -5.87 D 1 D 0.984 D 0.001 0.807 D 0.99807 0.706298 . . 2.532 0.655 6.052 -0.3039 -0.859 "Uncertain significance PVS1=0 PS=[0, 0, 0, 0, 0] PM=[1, 0, 0, 0, 0, 0, 0] PP=[0, 0, 0, 0, 1]" "Verdura.Brain,Brain : a journal of neurology,138,2347,2015.PubMed_ID:26063658"

chr10 124249235 124249235 C T Het snv PASS 61 0.53 115 "2049,0,1842" . . . NM_002775.4(HTRA1): c.777+93C>T HTRA1 5654 NM_002775.4 c.777+93C>T NP_002766.1 . + . intron g.124249234C>T . intron3 905+93 . 777+93 interior_intron . . 10q26.13 "Macular degeneration, age-related, neovascular type" 602194 AR 600142(MK)|610149(SK) P . . . . . . . . . . . . . Cerebral autosomal recessive arteriopathy with subcortical infarcts and leukoencephalopathy (CARASIL) AR General Cardiovascular; Dermatologic; Neurologic 17053108; 17053109; 17884985; 17568988; 18511946; 19387015; 20437615; 21115960; 21482952; 22900900 . . . . . . 0.7824 rs2239586 . . . . . . . . . . . . . . . . . . . . . . . . . . . . . 1196 0.238818 0.35922 0.43333 0.3591 0.21749084 0.185 dbsnp.98:rs2239586 . . . . . . . . . . . . . . . . . . . . . . . . . . . . . . . . . . 0.53 -0.15 0.545 0.0442 0.271 . .

chr10 124249309 124249309 G A Het snv PASS 29 0.49 59 "1017,0,1075" . . . NM_002775.4(HTRA1): c.777+167G>A HTRA1 5654 NM_002775.4 c.777+167G>A NP_002766.1 . + . intron g.124249308G>A . intron3 905+167 . 777+167 interior_intron . . 10q26.13 "Macular degeneration, age-related, neovascular type" 602194 AR 600142(MK)|610149(SK) P . . . . . . . . . . . . . Cerebral autosomal recessive arteriopathy with subcortical infarcts and leukoencephalopathy (CARASIL) AR General Cardiovascular; Dermatologic; Neurologic 17053108; 17053109; 17884985; 17568988; 18511946; 19387015; 20437615; 21115960; 21482952; 22900900 . . . . . . 0.7828 rs2239587 . . . . . . . . . . . . . . . . . . . . . . . . . . . . . 1193 0.238219 0.35922 0.43333 0.3591 0.21703297 0.185 dbsnp.98:rs2239587 . . . . . . . . . . . . . . . . . . . . . . . . . . . . . . . . . . 2.19 0.655 2.181 0.0283 0.187 . .

chr10 124266500 124266500 C T Het snv PASS 75 0.52 144 "2415,0,2196" . . . NM_002775.4(HTRA1): c.972+99C>T HTRA1 5654 NM_002775.4 c.972+99C>T NP_002766.1 . + . intron g.124266499C>T . intron4 1100+99 . 972+99 interior_intron . . 10q26.13 "Macular degeneration, age-related, neovascular type" 602194 AR 600142(MK)|610149(SK) P . . . . . . . . . . . . . Cerebral autosomal recessive arteriopathy with subcortical infarcts and leukoencephalopathy (CARASIL) AR General Cardiovascular; Dermatologic; Neurologic 17053108; 17053109; 17884985; 17568988; 18511946; 19387015; 20437615; 21115960; 21482952; 22900900 . . . . . . 0.6763 rs2672582 . . . . . . . . . . . . . . . . . . . . . . . . . . . . . 1552 0.309904 0.42233 0.30476 0.3859 0.32417582 0.25 dbsnp.100:rs2672582 . . . . . . . . . . . . . . . . . . . . . . . . . . . . . . . . . . -0.628 -4.114 -0.665 0.2212 0.763 . .

chr10 124267087 124267087 G A Het snv PASS 31 0.48 65 "1099,0,1225" . . . NM_002775.4(HTRA1): c.1005+169G>A HTRA1 5654 NM_002775.4 c.1005+169G>A NP_002766.1 . + . intron g.124267086G>A . intron5 1133+169 . 1005+169 interior_intron . . 10q26.13 "Macular degeneration, age-related, neovascular type" 602194 AR 600142(MK)|610149(SK) P . . . . . . . . . . . . . Cerebral autosomal recessive arteriopathy with subcortical infarcts and leukoencephalopathy (CARASIL) AR General Cardiovascular; Dermatologic; Neurologic 17053108; 17053109; 17884985; 17568988; 18511946; 19387015; 20437615; 21115960; 21482952; 22900900 . . . . . . 0.6758 rs2672583 . . . . . . . . . . . . . . . . . . . . . . . . . . . . . 1551 0.309704 0.42718 0.30476 0.3869 0.3246337 0.25 dbsnp.100:rs2672583 . . . . . . . . . . . . . . . . . . . . . . . . . . . . . . . . . . -1.976 -1.338 -2.234 1.5472 1.8 . .

chr10 124268397 124268397 G A Het snv PASS 66 0.51 129 "2202,0,2427" . . . NM_002775.4(HTRA1): c.1120+111G>A HTRA1 5654 NM_002775.4 c.1120+111G>A NP_002766.1 . + . intron g.124268396G>A . intron6 1248+111 . 1120+111 interior_intron . . 10q26.13 "Macular degeneration, age-related, neovascular type" 602194 AR 600142(MK)|610149(SK) P . . . . . . . . . . . . . Cerebral autosomal recessive arteriopathy with subcortical infarcts and leukoencephalopathy (CARASIL) AR General Cardiovascular; Dermatologic; Neurologic 17053108; 17053109; 17884985; 17568988; 18511946; 19387015; 20437615; 21115960; 21482952; 22900900 . . . . . . 0.9472 rs79778361 . . . . . . . . . . . . . . . . . . . . . . . . . . . . . 211 0.0421326 0.15049 0.2 0.1667 0.05265568 0.009 dbsnp.131:rs79778361 . . . . . . . . . . . . . . . . . . . . . . . . . . . . . . . . . . -1.232 -0.735 -1.181 0.1598 0.639 . .

chr10 124268401 124268401 C G Het snv PASS 62 0.52 120 "2062,0,2341" . . . NM_002775.4(HTRA1): c.1120+115C>G HTRA1 5654 NM_002775.4 c.1120+115C>G NP_002766.1 . + . intron g.124268400C>G . intron6 1248+115 . 1120+115 interior_intron . . 10q26.13 "Macular degeneration, age-related, neovascular type" 602194 AR 600142(MK)|610149(SK) P . . . . . . . . . . . . . Cerebral autosomal recessive arteriopathy with subcortical infarcts and leukoencephalopathy (CARASIL) AR General Cardiovascular; Dermatologic; Neurologic 17053108; 17053109; 17884985; 17568988; 18511946; 19387015; 20437615; 21115960; 21482952; 22900900 . . . . . . 0.6648 rs2672585 . . . . . . . . . . . . . . . . . . . . . . . . . . . . . 1594 0.318291 0.42233 0.29524 0.3839 0.33562271 0.296 dbsnp.100:rs2672585 . . . . . . . . . . . . . . . . . . . . . . . . . . . . . . . . . . -0.479 -0.14 -0.481 0.0104 0.071 . .

chr10 124268540 124268540 G C Hom snv PASS 18 1 18 "687,53,0" . . . NM_002775.4(HTRA1): c.1120+254G>C HTRA1 5654 NM_002775.4 c.1120+254G>C NP_002766.1 . + . intron g.124268539G>C . intron6 1248+254 . 1120+254 interior_intron . MIRb|SINE|MIR|-|150 10q26.13 "Macular degeneration, age-related, neovascular type" 602194 AR 600142(MK)|610149(SK) P . . . . . . . . . . . . . Cerebral autosomal recessive arteriopathy with subcortical infarcts and leukoencephalopathy (CARASIL) AR General Cardiovascular; Dermatologic; Neurologic 17053108; 17053109; 17884985; 17568988; 18511946; 19387015; 20437615; 21115960; 21482952; 22900900 . . . . . . 0.05647 rs2736925 . . . . . . . . . . . . . . . . . . . . . . . . . . . . . 4765 0.951478 1 1 0.999 0.94368132 0.935 dbsnp.100:rs2736925 . . . . . . . . . . . . . . . . . . . . . . . . . . . . . . . . . . 0.111 -0.12 0.093 0.2382 0.793 . .

chr10 124269818 124269818 C G Het snv PASS 90 0.53 169 "3063,0,2691" . . . NM_002775.4(HTRA1): c.1178+149C>G HTRA1 5654 NM_002775.4 c.1178+149C>G NP_002766.1 . + . intron g.124269817C>G . intron7 1306+149 . 1178+149 interior_intron . . 10q26.13 "Macular degeneration, age-related, neovascular type" 602194 AR 600142(MK)|610149(SK) P . . . . . . . . . . . . . Cerebral autosomal recessive arteriopathy with subcortical infarcts and leukoencephalopathy (CARASIL) AR General Cardiovascular; Dermatologic; Neurologic 17053108; 17053109; 17884985; 17568988; 18511946; 19387015; 20437615; 21115960; 21482952; 22900900 . . . . . . 0.9472 rs76357476 . . . . . . . . . . . . . . . . . . . . . . . . . . . . . 210 0.0419329 0.15049 0.2 0.1657 0.05265568 0.009 dbsnp.131:rs76357476 . . . . . . . . . . . . . . . . . . . . . . . . . . . . . . . . . . -1.429 -0.258 -1.452 0.0316 0.206 . .

chr10 124271589 124271589 G A Hom snv PASS 388 1 388 "15758,1178,0" . . . NM_002775.4(HTRA1): c.1274+8G>A HTRA1 5654 NM_002775.4 c.1274+8G>A NP_002766.1 . + . splice g.124271588G>A . intron8 1402+8 . 1274+8 interior_intron . . 10q26.13 "Macular degeneration, age-related, neovascular type" 602194 AR 600142(MK)|610149(SK) P . germline Macular_degeneration 299054 "criteria_provided,_single_submitter" Likely_benign . . . . . . . Cerebral autosomal recessive arteriopathy with subcortical infarcts and leukoencephalopathy (CARASIL) AR General Cardiovascular; Dermatologic; Neurologic 17053108; 17053109; 17884985; 17568988; 18511946; 19387015; 20437615; 21115960; 21482952; 22900900 . . . . . . 0.001377 rs2672586 121365 0.999843 4327 4327 PASS 15276 0.998301 246190 0.999838 33572 0.999702 9850 1 17248 1 22284 1 111692 0.999964 5486 1 30782 1 4398 0.998184 12998 0.999385 8600 1 5005 0.999401 1 1 1 0.99916295 0.991 dbsnp.100:rs2672586 . . . . . . . . . . . . . . . . . . . . . . . . . . . . . . . . . . -1.648 -0.361 -0.858 -0.847 -1.376 . .

chr10 124271595 124271595 G A Het snv LowQualFilter 240 0.66 366 "6747,0,3010" . . . NM_002775.4(HTRA1): c.1274+14G>A HTRA1 5654 NM_002775.4 c.1274+14G>A NP_002766.1 . + . intron g.124271594G>A . intron8 1402+14 . 1274+14 interior_intron . . 10q26.13 "Macular degeneration, age-related, neovascular type" 602194 AR 600142(MK)|610149(SK) P . germline Macular_degeneration 299055 "criteria_provided,_single_submitter" Likely_benign . . . . . . . Cerebral autosomal recessive arteriopathy with subcortical infarcts and leukoencephalopathy (CARASIL) AR General Cardiovascular; Dermatologic; Neurologic 17053108; 17053109; 17884985; 17568988; 18511946; 19387015; 20437615; 21115960; 21482952; 22900900 . . . . . . 0.3949 rs2272599 74133 0.611255 3725 1696 PASS 10830 0.708213 148365 0.602904 14604 0.434979 6138 0.623274 10785 0.625362 13354 0.600234 69305 0.620946 3386 0.617433 19963 0.648571 3084 0.699955 8462 0.650623 5378 0.625349 3145 0.627995 0.57767 0.67619 0.6071 0.60262864 0.626 dbsnp.100:rs2272599 . . . . . . . . . . . . . . . . . . . . . . . . . . . . . . . . . . -3.434 -0.361 -3.378 0.3788 0.996 . .

chr10 124273671 124273671 C T Het snv PASS 184 0.47 390 "5058,0,5646" . . . NM_002775.4(HTRA1): c.1275-36C>T HTRA1 5654 NM_002775.4 c.1275-36C>T NP_002766.1 . + . intron g.124273670C>T . intron8 1403-36 . 1275-36 interior_intron . . 10q26.13 "Macular degeneration, age-related, neovascular type" 602194 AR 600142(MK)|610149(SK) P . . . . . . . . . . . . . Cerebral autosomal recessive arteriopathy with subcortical infarcts and leukoencephalopathy (CARASIL) AR General Cardiovascular; Dermatologic; Neurologic 17053108; 17053109; 17884985; 17568988; 18511946; 19387015; 20437615; 21115960; 21482952; 22900900 . . . . . . 0.741 rs2293871 26788 0.220703 2878 818 PASS 2165 0.141466 54409 0.220993 6313 0.187988 2220 0.225426 7459 0.432456 2968 0.133166 20617 0.184624 1170 0.213426 11497 0.373522 599 0.135951 2235 0.171844 1636 0.190233 1308 0.261182 0.39806 0.50476 0.4087 0.32719151 0.25 dbsnp.100:rs2293871 . . . . . . . . . . . . . . . . . . . . . . . . . . . . . . . . . . -0.286 -1.327 -0.088 . . . .
